# Arabinoxylan rice bran (MGN-3/Biobran) enhances radiotherapy in animals bearing Ehrlich ascites carcinoma^†^

**DOI:** 10.1093/jrr/rrz055

**Published:** 2019-09-16

**Authors:** Nariman K Badr El-Din, Said K Areida, Kvan O Ahmed, Mamdooh Ghoneum

**Affiliations:** 1 Department of Zoology, Faculty of Science, University of Mansoura, Mansoura, Egypt; 2 Al-Qalam University, Kerkuk, At-Ta'mim, Iraq; 3 Department of Surgery, Drew University of Medicine and Science, Los Angeles, CA, USA

**Keywords:** radiotherapy, Biobran, apoptosis, *in vivo*

## Abstract

This study examines the ability of arabinoxylan rice bran (MGN-3/Biobran) to enhance the anti-cancer effects of fractionated X-ray irradiation of Ehrlich solid tumor-bearing mice. Swiss albino mice bearing tumors were exposed to the following: (i) Biobran treatment (40 mg/kg/day, intraperitoneal injections) beginning on day 11 post-tumor cell inoculation until day 30; (ii) ionizing radiation (Rad) 2 Gy at three consecutive doses on days 12, 14 and 16; or (iii) Biobran + Rad. Final tumor weight was suppressed by 46% for Biobran, 31% for Rad and 57% for the combined treatment (Biobran + Rad) relative to control untreated mice. Biobran and Rad also arrested the hypodiploid cells in the sub-G1-phase, signifying apoptosis by +102% and +85%, respectively, while the combined treatment induced apoptosis by +123%, with similar results in the degree of DNA fragmentation. Furthermore, Biobran + Rad upregulated the relative gene expression and protein level of p53 and Bax in tumor cells, down-regulated Bcl-2 expression, and increased the Bax/Bcl-2 ratio and caspase-3 activity, with the combined treatment greater than for either treatment alone. Additionally, the combined treatment modulated the decrease in body weight, the increase in liver and spleen weight, and the elevation of liver enzymes aspartate aminotransferase, alanine aminotransferase and gamma-glutamyl transferase to be within normal values. We conclude that Biobran enhances radiation therapy-induced tumor regression by potentiating apoptosis and minimizing toxicities related to radiation therapy, suggesting that Biobran may be useful in human cancer patients undergoing radiotherapy and warranting clinical trials.

## INTRODUCTION

Cancer is currently the second leading cause of death worldwide [1]. It develops from the uncontrolled growth of a proliferating cellular clone due to acquisition of self-sufficiency in growth signals, insensitivity to anti-growth signals, the ability to evade apoptosis, and limitless replicative potential [2]. Cancer is one of the most feared causes of morbidity and mortality all over the world—a significant proportion of this burden is borne by developing countries which report nearly 70% of the world’s cancer deaths [1, 3–4].

Radiotherapy continues to be one of the main treatment modalities for different types of cancers. However, the effectiveness of radiotherapy is limited by cancer cells’ radioresistance [5, 6], requiring a very high dose to successfully kill tumors. Such doses would ultimately cause undesirable complications to the surrounding normal tissues and to the distant organs of the cancer patient. This poses a severe limitation on radiotherapy [7]. It is a major challenge to radiation oncologists and researchers to develop alternative approaches that can be used to enhance the antitumor effects of radiation treatment, minimize radiation dosage, and evade the detrimental consequences of radiotherapy [8].

Researchers have begun to focus on radio-sensitizers, products that can lower the radiation dose response threshold of cancer cells without enhancing the radio-sensitivity of normal cells. Radio-protective and radio-sensitizing actions have been shown to be exerted by a variety of natural products such as medicinal plants and herbs and by polyphenol products derived from berries and seeds. These include the plants Aegle marmelos, Aloe arborescens, Angelica sinensis and Aphanamixis polystachya [9]; herbal medicines such as eugenol, ellagic acid, Triphala, tocopherol succinate and embelin [10–12]; and the polyphenol products resveratrol (from berries) and (-)-gossypol (from cottonseed) [13, 14]. Intervention with radiosensitizers aims to increase the efficacy of radiotherapy treatment and decrease its side effects by improving cancer cell killing through apoptosis, reducing treatment-resistance in cancer cells, detoxifying the body, decreasing weight loss and malnutrition, and improving the quality of life. Given that earlier studies on the natural product MGN-3/Biobran (derived from rice bran) have demonstrated it to have potential tumor inhibitory effects [15] and potent chemo-sensitizing effects [16, 17], we set out in the current study to determine the potential of Biobran for enhancing the therapeutic efficacy of X-ray irradiation (Rad) against solid tumors.

Biobran is obtained by reacting rice bran hemicellulose with multiple carbohydrate hydrolyzing enzymes from Shiitake mushrooms [18]. Biobran has been reported to be a potent activator of human NK cells in healthy subjects and in cancer patients [19–21]. In addition, *in vivo* studies on animals bearing tumors revealed that Biobran has a potent tumor inhibitory effect by a mechanism involving induction of tumor cell apoptosis [15]. The induction of apoptosis may control the response of tumor cells to treatment with anti-cancer drugs [22]. Furthermore, Biobran has been shown to be a chemo-sensitizing agent that possesses great potential for adjuvant therapy in the treatment of cancer: Biobran sensitizes human leukemic HUT 78 cells to anti-CD95 antibody-induced apoptosis [23], sensitizes human breast cancer MCF-7 cells and murine metastatic breast cancer 4T1 to paclitaxel *in vitro* [16] and sensitizes breast adenocarcinoma to paclitaxel *in vivo* [17]. The current study investigates the ability of Biobran to enhance the effects of Rad on Ehrlich solid carcinoma cells and investigates the underlying molecular mechanism of its action.

## MATERIALS AND METHODS

### MGN-3/Biobran

Biobran is a denatured hemicellulose obtained by reacting rice bran hemicellulose with multiple carbohydrate hydrolyzing enzymes obtained from Shiitake mushrooms. The main chemical structure of Biobran is arabinoxylan with an arabinose polymer in its side chain and a xylose in its main chain [18]. Biobran was provided by Daiwa Pharmaceutical Co. Ltd. (Tokyo, Japan) and was freshly prepared by dissolving in 0.9% saline solution and administered by intraperitoneal injections (i.p.) at a dose of 40 mg/kg body weight (BW)/day, 5 times/week to mice with solid Ehrlich carcinoma. Treatment began on day 11 post-tumor cells inoculation and ended on day 30 for a total of 15 injections.

### X-ray irradiation

X-ray irradiation was performed at the oncology center, Mansoura University, Egypt using a linear accelerator machine, LINAC. Animals received whole body X-ray ionizing radiation at a dose level 6 Gray (Gy) divided into three fractionated doses (2 Gy each with a dose rate of 0.85 Gy/min) on days 12, 14 and 16 post-tumor cell inoculation.

### Preparation of Ehrlich ascites carcinoma cells and tumor transplantation

Ehrlich ascites carcinoma (EAC) cells were generously supplied by the National Cancer Institute, Cairo University, (Cairo, Egypt). Cells were maintained by weekly i.p. transplantation at a dose of 2.5×10^6^ cells in female Swiss albino mice. In this experiment, solid tumors were produced by intramuscular injection of 0.2 ml EAC cells (2.5×10^6^ cells) in the right thigh muscle of the mice. Tumor cell viability was determined to be 95% using the trypan blue dye exclusion method. Mice with solid tumor mass (~300 mm^3^) that developed within 9 days post-inoculation were used in the study.

### Animals

A total of 60 female 2-month-old Swiss albino mice weighing 22 ± 2 g were used in this study. The mice were purchased from the National Cancer Institute, Cairo University, (Cairo, Egypt) and were housed at constant temperature (24^◦^C ± 2^◦^C) 75^◦^F at 10% relative humidity, and alternating 12-h light/dark cycles in our animal research facility. Mice were accommodated for 1 week prior to experiments. Animals were provided with water *ad libitum* and standard food pellets. All animal experiments were conducted with the approval of the University of Mansoura, Egypt, and animal protocols were in compliance with their Guide for the Care and Use of Laboratory Animals.

### Experimental design

Mice were randomly divided into six groups as follows: (i) Untreated Control group (8 mice); (ii) Control Biobran group: control mice treated with Biobran alone (8 mice); (iii) Inoculated (Inocul) Control group: mice bearing tumor receiving intratumoral injections of PBS (11 mice); (iv) Inocul Biobran group: mice bearing tumor receiving Biobran [40 mg/kg BW/day] (11 mice); (v) Inocul Rad (irradiated) group: mice bearing tumor subjected to whole body Rad at a dose level (6 Gy) divided into three fractionated doses (11 mice); and (vi) Inocul Biobran + Rad group: mice-bearing tumor treated with Biobran (40 mg/kg BW/day) and subjected to whole body Rad at a dose level (6 Gy) divided into three fractionated doses (11 mice).

### Sample collections

At the experimental endpoint (day 30), animals were fasted for 16 h and anesthetized using diethyl ether. Using vacuum tubes, blood was drawn from the abdominal aorta and left to clot at room temperature. The serum was then separated by centrifugation at 3000 r.p.m. for 20 min and stored until assayed. Serum was used for the determination of liver function test parameters. Animals were dissected to obtain solid tumor, liver and spleen to perform various analyses. Parameters under investigation included BW, organ weight (liver and spleen), tumor volume (TV) and tumor weight (TW). Cell cycle progression, apoptosis and apoptotic protein regulators (p53, Bax, Bcl-2 and caspase-3) were determined using flow cytometry analysis, and relative gene expression (p53, Bax, Bcl-2) was assayed using real-time PCR. Tumor DNA damage was detected using gel electrophoresis and tumor apoptosis and necrosis were histochemically examined with florescence microscopy.

### Evaluation of body and organ weight

Mice were examined for final and net BWs at day 30 and compared with initial BWs. The net final BW = final BW−TW. BW gain was defined as the difference between the initial and the net final BW. Organ weight such as liver and spleen weights were examined at day 30 post-scarification of animals.

### Tumor growth

Tumor growth was determined by measuring TV and TW. Using digital Vernier calipers, time interval measurements of TV (3 days/week) were conducted from day 9 to day 30 post-EAC cell inoculation. Data collected were analyzed using the following formula to obtain tumor volume: TV (mm^3^) = 0.52*AB*^2^, where *A* is the minor axis and *B* is the major axis. Solid tumors were removed at the end of the experiment for TW determination, photographed and processed for the different analyses. For reference, the initial TVs on day 9 for the Inocul Control, Inocul Biobran, Inocul Rad and Inocul Biobran + Rad groups were 342±9.1, 367±9.7, 388±12.3 and 351±8.0 mm^3^, respectively.

### Flow cytometric analysis

#### Cell preparation

Tumor tissues were excised from EAC-bearing mice from each group, cut into pieces and rubbed through fine nylon gauze (40–50 mesh count/cm; HD 140 Zuricher Buteltuch fabrik AG, Zürich, Switzerland). Samples were washed through the gauze with Tris-ethylenediaminetetraacetic acid (Tris-EDTA) buffer at pH 7.5 [3.029 g of 0.1 M Tris-(hydroxymethyl aminomethane), 1.022 g of 0.07 M HCl and 0.47 g of 0.005 M Tris-EDTA]. Cells were suspended in sterile PBS, centrifuged for 5 min at 200–300 × g, resuspended in PBS (cell density ~1×10^6^ cells/ml) and fixed in 70% ice-cold ethanol mixed with PBS and stored at −20^◦^C until analyzed.

#### Cell cycle analysis by propidium iodide

Tumor cell suspensions were centrifuged and cell pellets were resuspended in 1 ml of propidium iodide (PI) solution and incubated for 30 min in the dark. Subsequent assays were performed using flow cytometry. Data analysis was conducted using the DNA analysis program MODFIT (Verity Software House, Inc., Topsham, ME, USA). For each sample, the software calculated the coefficient of variation around the G0/G1 peak and the percentage of cells in each phase (G0/G1, S and G2/M) of the DNA cell cycle. An aneuploid cell population was considered present if a distinct peak, in addition to the G1 diploid peak, deviated more than 10% from the diploid internal standard, or if the G1 itself deviated more than 10% from a corresponding G2/M peak. The apoptosis index (AI)/proliferation index (PrI) ratio was calculated [24].

#### Expression of apoptosis related protein

Mouse antibodies against p53(sc-7480), Bax(sc-7480), Bcl-2(sc-7382), caspase-3(sc-7272) and other reagents were purchased from Santa Cruz Biotechnology, Inc., Dallas, TX, USA. Secondary antibodies were available as fluorescein [fluorescein isothiocyanate (FITC)] conjugates for flow cytometry. Tumor cells (1×10^6^) from mice treated with Biobran and/or Rad were incubated with the appropriate antibody for 1 h at room temperature followed by FITC-conjugated goat-anti-rabbit antibody. Cells were washed thoroughly with phosphate-buffered saline and bovine serum albumin and analyzed on a flow cytometer (Becton Dickinson, San Jose, CA, USA). A total of 20 000 cells were acquired for analysis using CellQuest software, and histogram plots of FITC-fluorescence vs counts in logarithmic fluorescence intensity were used to obtain mean values.

### Reverse transcription-polymerase chain reaction analysis of apoptotic regulators

Following the manufacturer’s instructions, total RNA extraction was performed using a GF-TR-050 Total RNA Extraction Kit (Vivantis Technologies SDN. BHD., Malaysia). The total RNA was reverse transcribed into cDNA using FastQuant RT Kit by (Tiangen Biotech (Beijing) Co., Ltd). The kit contained gDNase that removed genomic DNA by incubation at 42°C for 3 min to protect the total RNA analysis from genomic DNA interference. Real-time reverse transcription-polymerase chain reaction (RT-PCR) was performed using Maxima SYBR Green qPCR Master Mix (2×) Kit (Thermo Scientific). Reaction conditions and data analysis were used per the manufacturer’s instructions: 5 μl of cDNA in a total volume of 25 μl containing 12.5 μl Maxima SYBR Green qPCR Master Mix (2×), 0.3 μMol forward primer, 0.3 μMol reverse primer (primers are shown in Table 1), 10 nM/100n ROX Solution, and brought up to 25 μl with nuclease-free water. Thermal cycling conditions were 95°C for 10 min, followed by 40 cycles of 95°C for 15 s, 58°C for 30 s, and 60°C for 30 s. Reactions were run with a PIKO REAL 96 Real-Time PCR system, (Thermo Scientific). Differences in gene expression between groups were determined using the ΔΔC cycle time (Ct) method [25], and were normalized against the glyceraldehyde-3-phosphate dehydrogenase (GAPDH) house-keeping gene. Data were expressed as relative mRNA levels as compared with the level of the Inocul Control group.

**Table 1.** The primers used for the amplification of the respective genes in the real-time RT-PCR. p53, Bcl-2 and GAPDH are designed by (Vivantis, Malaysia), Bax by (Metabion, Germany)

Gene	Forward primer	Reverse primer
**p53**	**5-GTC ACA GCA CAT GAC GGA GG-3**	**5-CTG TGG CGA AAA GTC TGC CT-3**
**Bax**	**5-ATG CTC CAC CAA GAA GCT GA-3**	**5-AGC AAT CAT CCT CTG CAG CTC C-3**
**Bcl-2**	**5-GCG TCA ACA GGG AGA TGT CA-3**	**5-GCA TGC TGG GGC CAT ATA GT-3**
**GAPDH**	**5-TGA TGG GTG TGA ACC ACG AG-3**	**5-GCC CTT CCA CAA TGC CAA AG-3**

### Detection of DNA damage by gel electrophoresis

#### DNA extraction

DNA extraction of tumor tissue (30 mg) was performed using a TIANamp Genomic DNA Kit (TIANGEN Biotech (Beijing) Co., Ltd) following the manufacturer’s instructions.

#### Gel electrophoresis

Standard gel electrophoresis in an agarose gel was used to separate DNA by size (e.g., length in base pairs) in order to visualize and purify DNA samples. The length of a DNA segment was determined using a DNA ladder. After running, the gel was analyzed by Gel analyzer Pro v3.1, which automatically detected lanes and bands. This was also analyzed quantitatively using ImageJ v1.48 software.

### Histochemical demonstration of apoptosis and necrosis within the tumor tissue

Apoptosis in tumor tissues of each group was determined using acridine orange–ethidium bromide co-staining, and examined under a fluorescence microscope [26].

#### Sample preparation

Tumor specimens were fixed in 10% buffered neutral formalin, dehydrated, and then embedded in paraffin. Sections of 5 μm were slide-mounted on positively charged slides and de-paraffinized by immersion in three changes of xylene for 5 min each. Tissue was then rehydrated by washing in graded alcohol, 3 min for each, after which they were rinsed in PBS three times.

#### Staining

The slides were immersed in a mixture of 100 μg/ml acridine orange and 100 μg/ml ethidium bromide freshly prepared in PBS. After cover-slipping the slides, apoptotic and necrotic cells were examined under a fluorescence microscope with 4′,6-diamidino-2-phenylindole-dihdrochloride (DAPI), FITC and Texas red filters (at 400 nm, 495 nm and 570 nm, respectively). Quantitative analysis was performed using ZEN 2011 blue edition imaging software.

### Liver function analysis

#### Determination of alanine aminotransferase and aspartate aminotransferase activity

Alanine aminotransferase (ALT) and aspartate aminotransferase (AST) activity in serum was assayed by a kinetic method described by the International Federation of Clinical Chemistry (IFCC) using a diagnostic kit supplied by ELITech Clinical System, France. The activity value of the transmittance obtained was calculated using the following formula:

Activity(U/L)=ΔA/min×1746

#### Determination of gamma-glutamyl transferase activity

Gamma-glutamyl transferase (GGT) activity in serum was assayed using a diagnostic kit supplied by ELITech Clinical System, France. The activity value of the transmittance obtained was calculated using the following formula:

Activity(U/L)=ΔA/min×2211

### Statistical analysis

Results were expressed as means ± SE. Statistical significance was calculated using one-way analysis of variance (ANOVA) followed by *post hoc* tests for multiples comparisons. All the statistical analysis was carried out with the use of SPSS 17 software. Differences were considered significant at *P* ≤ 0.05.

## RESULTS

### Body weight

**Fig. 1.**
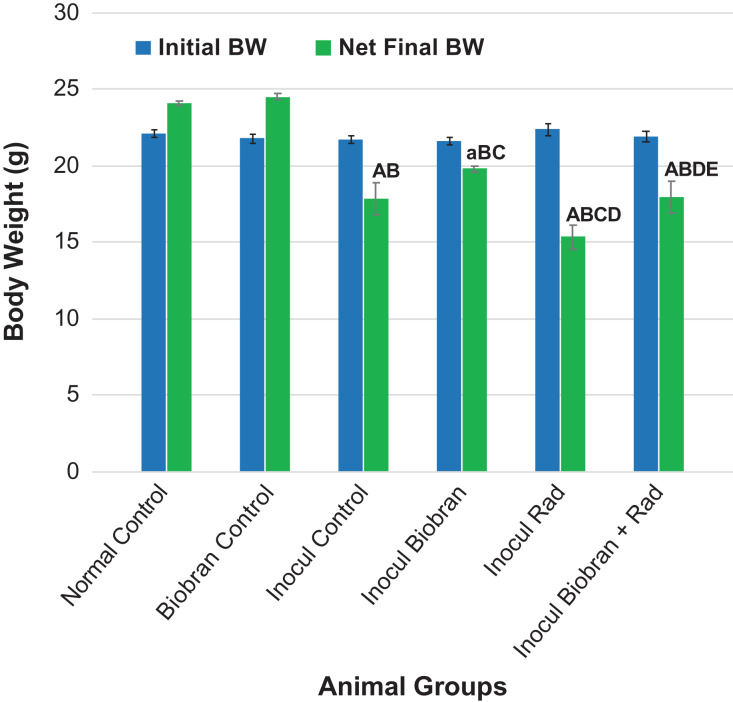
Effect of Biobran and/or X-ray irradiation on body weight change (g). Each value represents the mean ± SE of 8–11 mice. Number of mice/group for (Initial, Net Final BW): Normal Control (8, 8), Normal Biobran (8, 8), Inocul Control (11, 10), Inocul Biobran (11, 11), Inocul Rad (11, 6), Inocul Biobran + Rad (11, 10). ^a,A^Significantly different from the net final BW of Normal Control group at *P* < 0.05, *P* < 0.01 levels. ^B^Significantly different from the net final BW of Normal Biobran group at *P* < 0.01 level. ^C^Significantly different from the net final BW of Inocul Control group at *P* < 0.01 level. ^D^Significantly different from the net final BW of Inocul Biobran group at *P* < 0.01 level. ^E^Significantly different from the net final BW of Inocul Rad group at *P* < 0.01 level.

BW of the different experimental groups was recorded weekly during the experiment. Figure 1 shows the initial and the net final BWs of animals under different treatment conditions. Initial BW was comparable between groups; however, post-treatment (net final) BWs exhibited marked differences for inoculated animals. Relative to initial values, inoculated control (Inocul Control) animals experienced an 18% decrease in BW. Treatment with Biobran minimized the BW loss to only 4.1%. Rad showed a 31.2% decrease in BW, while the combination of Biobran + Rad markedly reduced the BW loss, recording a decrease of 17.9% as compared with the Inocul Control animals.

### Tumor volume

**Fig. 2.**
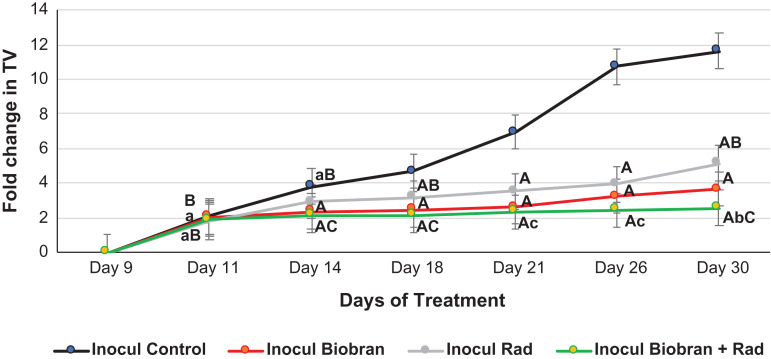
Effect of Biobran and/or X- ray irradiation on tumor volume. Data are expressed as mean ± SE. ^a,A^Significantly different from Inocul Control group at *P* < 0.05, *P* < 0.01 levels, respectively at the corresponding time point. ^b,B^Significantly different from Inocul Biobran group at *P* < 0.05, *P* < 0.01 levels, respectively at the corresponding time point. ^c,C^Significantly different from Inocul Rad group at *P* < 0.05, *P* < 0.01 levels, respectively at the corresponding time point.

**Fig. 3.**
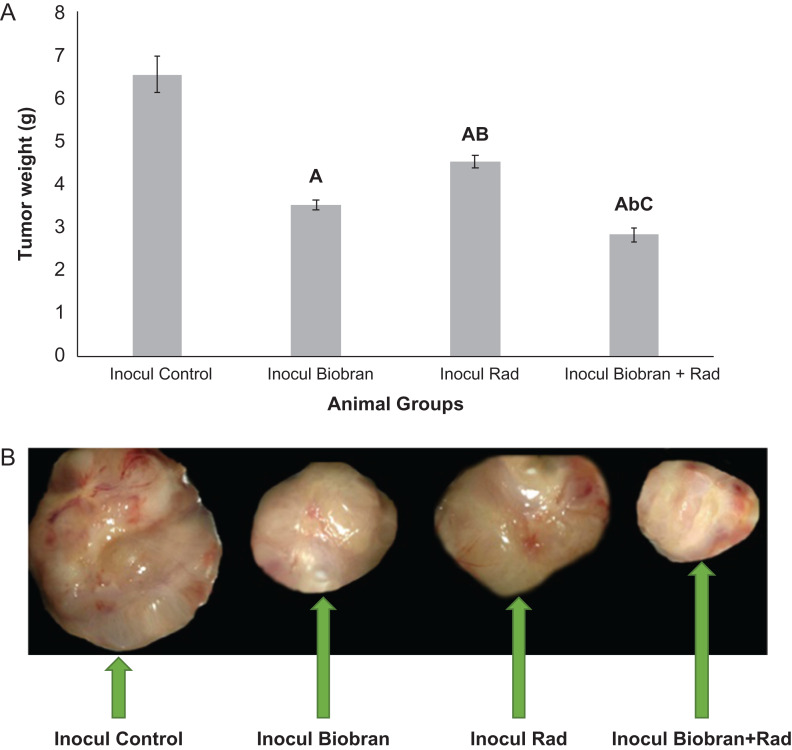
(A) Effect of Biobran and/or Rad on tumor weight. Each value represents the mean ± SE. Number of mice/group; Inocul Control (10), Inocul Biobran (11), Inocul Rad (6), Inocul Biobran + Rad (10). (B) Representative photograph of tumor regression after treatment with Biobran and/or X-ray irradiation after 30 days. ^A^Significantly different from Inocul Control group at *P* ≤ 0.01 level. ^b,B^Significantly different from Inocul Biobran group at *P* ≤ 0.05, *P* ≤ 0.01 levels, respectively. ^C^Significantly different from Inocul Rad group at *P* ≤ 0.01 level.

Figure 2 illustrates the effect of Biobran and/or Rad on the growth of EAC post-inoculation as defined by fold change in TV (final tumor/initial tumor). Biobran treatment resulted in continuous suppression of TV that reached 33.7%, *P* < 0.01, at day 14 (2.35-fold vs 3.81-fold for control) and maximized at 66.4%, *P* < 0.01, reduction at day 30 (3.65-fold vs 11.64-fold for control). Exposure to Rad caused a reduction in TV that reached 49.9%, *P* < 0.01, at day 30 (5.14-fold vs 11.64-fold for control). On the other hand, combinatorial treatment (Biobran + Rad) resulted in a profound retardation of TV that reached −42.0%, *P* < 0.01, at day 14 (2.15-fold vs 3.81-fold), and further increased to −77.3%, *P* < 0.01, at day 30 relative to Inocul Control mice (2.57-fold relative to 11.64-fold). These results are well illustrated in the photographs of tumor regression Fig. 3B.

### Tumor weight

On day 30, tumors were excised and weighed to evaluate the effect of different treatments on TW. As shown in Fig. 3A and B, treatments with Biobran caused a marked reduction in TW by 46.3% and exposure to Rad caused a 30.7% reduction. However, Rad suppressive effect was further increased in the presence of Biobran and reached 56.9% relative to the Inocul Control group.

### Cell cycle analysis

Cell cycle analysis was evaluated by flow cytometry of PI-stained cells as shown in Table 2. The hypodiploid cells in the sub-G1-phase were markedly increased in the groups treated with Biobran alone or Rad by 102% and 85% respectively relative to Inocul Control group. In contrast, combined treatment with Biobran + Rad significantly maximized the hypodiploid cells in the sub-G1-phase and reached 123% as compared with the Inocul Control group. Administration of Biobran or fractionated Rad to animals bearing tumor caused disruption of the tumor cell cycle status (G0/G1, S and G2/M phases) with no cell cycle arrest.

**Table 2.** Cell cycle progression in tumor tissues under different treatment conditions; each value represents the mean ± SE of five mice/group

Parameter	Inocul			
	Control	Biobran	Rad	Biobran + Rad
**Sub G1 (M1)**	**36.7 ± 1.44**	**74.1 ± 0.48** *	**67.7 ± 1.37** *^,^**	**81.9 ± 1.11** *^,^**^,^***
**% Change from Inocul Control group**	**-**	**+102 %**	**+85 %**	**+123 %**
**G0/G1 (M2)**	**42.8 ± 1.46**	**17.3 ± 0.82** *	**19.5 ± 1.08** *	**12.3 ± 0.62** *^,^**^,^***
**% Change from Inocul Control group**	**-**	**−60 %**	**−55 %**	**−71 %**
**S phase (M3)**	**12.6 ± 0.29**	**6.0 ± 0.41** *	**8.3 ± 0.44** *^,^**	**4.1 ± 0.37** *^,^**^,^***
**% Change from Inocul Control group**	**-**	**−52 %**	**−34 %**	**−68 %**
**G2/M (M4)**	**6.5 ± 0.30**	**2.4 ± 0.45** *	**3.9 ± 0.11** *^,^**	**1.9 ± 0.13** *^,^***
**% Change from Inocul Control group**	**-**	**−62 %**	**−40 %**	**−70 %**

*Significantly different from Inocul Control group at *P* ≤0.01 level.

**Significantly different from Inocul Biobran group at *P* ≤0.01 level.

***Significantly different from Inocul Rad group at *P* ≤0.01 level.

### Effect of Biobran and Rad on the apoptosis index/proliferation index ratio

**Fig. 4.**
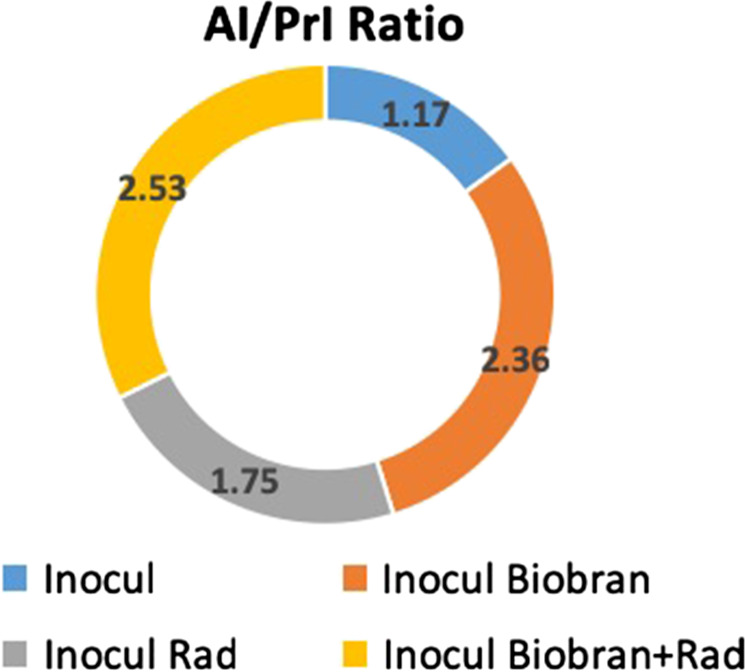
Effect of Biobran and Rad on the AI/PrI ratio. Each value represents the mean of five mice/group; SE for the Inocul Control, Inocul Biobran, Inocul Rad, and Inocul Biobran + Rad groups are 0.070, 0.219, 0.0359 and 0.099 respectively.

As shown in Figure 4, Biobran treatment alone increased the AI/PrI ratio 2-fold, and Rad treatment alone resulted in an increase in the AI/PrI ratio of 1.5-fold. The combination of Biobran + Rad maximized the AI/PrI ratio to reach 2.2-fold (*P < 0.01*) as compared with the Inocul Control group.

### Quantitative histochemical analysis of apoptosis and necrosis within the tumor tissue

**Fig. 5.**
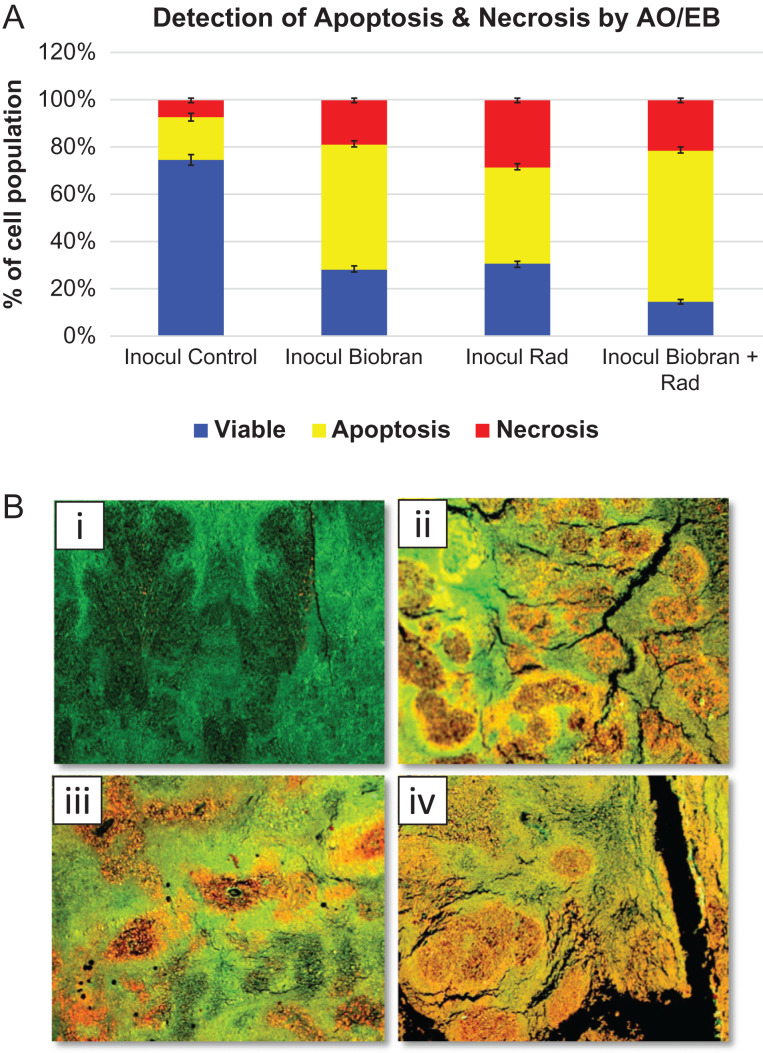
(A) Histochemical detection of % apoptosis and necrosis in tumor tissues of the different groups. Each value represents the mean ± SE of five tumor samples/group. (B) Representative fluorescent photomicrographs of sections of Inocul mice treated with Biobran and/or X-ray irradiation, stained with acridine orange and ethidium bromide showing apoptosis and necrosis. (i) untreated Inocul; (ii) Inocul Biobran; (iii) Inocul Rad; (iv) Inocul Biobran + Rad. Acridine orange and ethidium bromide. 50×. Viable cells: green color, apoptotic cells: yellow to light orange, necrotic cells: dark orange to red.

Quantitative histochemical detection of apoptosis/necrosis by acridine orange–ethidium bromide was carried out within the tumor tissue (Figures 5A and B). Tumor tissue of the untreated Inocul group recorded 74.5 ± 2.25% viable cells, 18.2 ± 1.68% apoptotic cells and 7.3 ± 1.4% necrotic cells. Tumor tissues of the Inocul Biobran group showed 28.2 ± 1.25% viable cells, 53.1 ± 1.21% apoptotic cells and 18.8 ± 0.96% necrotic cells. Tumor tissues of animals exposed to Rad recorded 30.3 ± 1.23% viable cells, 41.3 ± 1.22% apoptotic cells and 28.4 ± 0.89% necrotic cells. On the other hand, tumor tissues of animals treated with Biobran + Rad recorded 14.6 ± 0.93% viable cells, 64.0 ± 1.47% apoptotic cells and 21.4 ± 1.7% necrotic cells.

### Analysis of apoptotic regulators

**Fig. 6.**
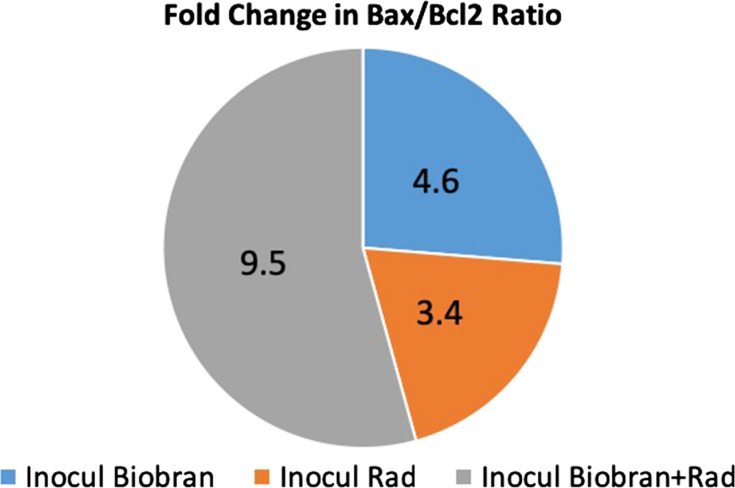
Fold change in Bax/Bcl-2 ratio in five tumor samples/group relative to the untreated Inocul Control. SE of mean values are available in Table 3.

In order to understand the mechanism by which Biobran enhanced the apoptotic effects of fractionated Rad, changes in the expression levels of apoptotic proteins were evaluated in tumor cells of the different groups as represented in Table 3. Significant upregulation in expression for p53, Bax and caspase-3 with a significant decrease in Bcl-2 expression was observed in Biobran + Rad groups relative to control and the effect was larger than either treatment alone. Changes in Bax/Bcl-2 ratio are shown in Table 3 and Fig. 6. Bax/Bcl-2 ratio was increased relative to control 3.4-fold for Rad alone and maximized at 9.5-fold for co-treatment.

**Table 3.** Effect of Biobran and/or Rad on apoptotic regulators in tumor; each value represents the mean ± SE of 5 tumor samples/group

Parameter	Inocul			
	Control	Biobran	Rad	Biobran + Rad
**p53 expression**	**15.67 ± 0.41**	**33.50±0.26** *	**31.15 ± 0.16** *^,^**	**45.05 ± 0.23** *^,^**^,^***
**% Change from Inocul Control group**	**-**	**+113.78 %**	**+98.78 %**	**+187.49 %**
**Bax expression**	**12.34 ± 0.12**	**26.42 ± 0.77** *	**23.78 ± 0.27** *^,^**	**37.03 ± 0.34** *^,^**^,^***
**% Change from Inocul Control group**	**-**	**+114.1 %**	**+92.7 %**	**+200.08 %**
**Bcl-2 expression**	**51.12 ± 0.35**	**23.86 ± 0.37** *	**29.14 ± 0.11** *^,^**	**16.24 ± 0.22** *^,^**^,^***
**% Change from Inocul Control group**	**-**	**−53.32 %**	**−42.99 %**	**−68.23 %**
**Bax/Bcl-2 ratio**	**0.242 ± 0.003**	**1.108 ± 0.036** *	**0.816 ± 0.008** *^,^**	**2.283 ± 0.049** *^,^**^,^***
**% Change from Inocul Control group**	**-**	**+358.9 %**	**+237.9%**	**+845.3 %**
**Caspase-3 expression**	**23.89 ± 1.86**	**53.32 ± 1.76** *	**38.64 ± 2.08** *^,^**	**71.6 ± 1.74** *^,^**^,^***
**% Change from Inocul Control group**	**-**	**+123.22 %**	**+61.78 %**	**+199.77 %**

*Significantly different from the corresponding Inocul Control group at *P* ≤ 0.01 level.

**Significantly different from the corresponding Inocul Biobran group at *P* ≤ 0.01 level.

***Significantly different from the corresponding Inocul Rad group at *P* ≤ 0.01 level.

### Analysis of p53, Bax and Bcl-2 gene expression by RT-PCR

Detection of p53, Bax and Bcl-2 gene expression in tumor tissues of the different groups by quantitative RT-PCR is shown in Table 4. Treatment by Biobran alone of tumor-bearing mice significantly upregulated p53 and Bax gene expression by 149.4 and 133.2% respectively, and downregulated Bcl-2 gene expression by 65.2% as compared with the Inocul Control group.

**Table 4.** Effect of Biobran and/or Rad on relative gene expression of p53' Bax and Bcl-2 in tumor tissues as determined by RT-PCR. Relative gene expression was quantified with GAPDH as an internal control. Data were represented as % increase or decrease relative to the levels of Inocul Control group. The relative gene expression of Inocul Control group was defined as 1. Each value represents the mean ± SE of five tumor samples/group.

Parameter	Inocul			
	Control	Biobran	Rad	Biobran + Rad
**p53 gene**	**1**	2.49 ± 0.15*	**1.58 ± 0.14** ^†,^**	**3.84 ± 0.23** *^,^**^,^***
**% Change from Inocul Control group**	**-**	**+149.4**	**+58.8**	**+284.9**
**Bax gene**	**1**	**2.33 ± 0.19** *	**1.43 ± 0.16** **	**3.44 ± 0.43** *^,^**^,^***
**% Change from Inocul Control group**	**-**	**+133.2**	**+43.4**	**+244.1**
**Bcl-2 gene**	**1**	**0.348 ± 0.045** *	**0.636 ± 0.052** *^,^**	**0.046 ± 0.014** *^,^**^,^***
**% Change from Inocul Control group**	**-**	**−65.2**	**−36.4**	**−95.4**

^†,^*Significantly different from Inocul Control group at *P* < 0.05, *P* < 0.01 levels.

**Significantly different from Inocul Biobran group at *P* < 0.01 level.

***Significantly different from Inocul Rad group at *P* < 0.01 level.

Exposure to Rad enhanced p53 and Bax expression by 58.8 and 43.4%, respectively, and decreased Bcl-2 expression by 36.4%, relative to the Inocul Control group. In contrast, treatment with Biobran + Rad further increased upregulation of p53 and Bax expression by 284.9 and 244.1% respectively, and Bcl-2 expression was markedly suppressed by 95.4% relative to the Inocul Control group.

### Detection of DNA damage by gel electrophoresis

To understand the mechanism of how Biobran enhanced the apoptotic effect of radiotherapy, the nuclear DNA fragmentation-based apoptosis approach, a characteristic hall-mark of apoptosis, was investigated by DNA gel electrophoresis using characteristic ladders of DNA fragmentation that signify apoptosis.

**Fig. 7.**
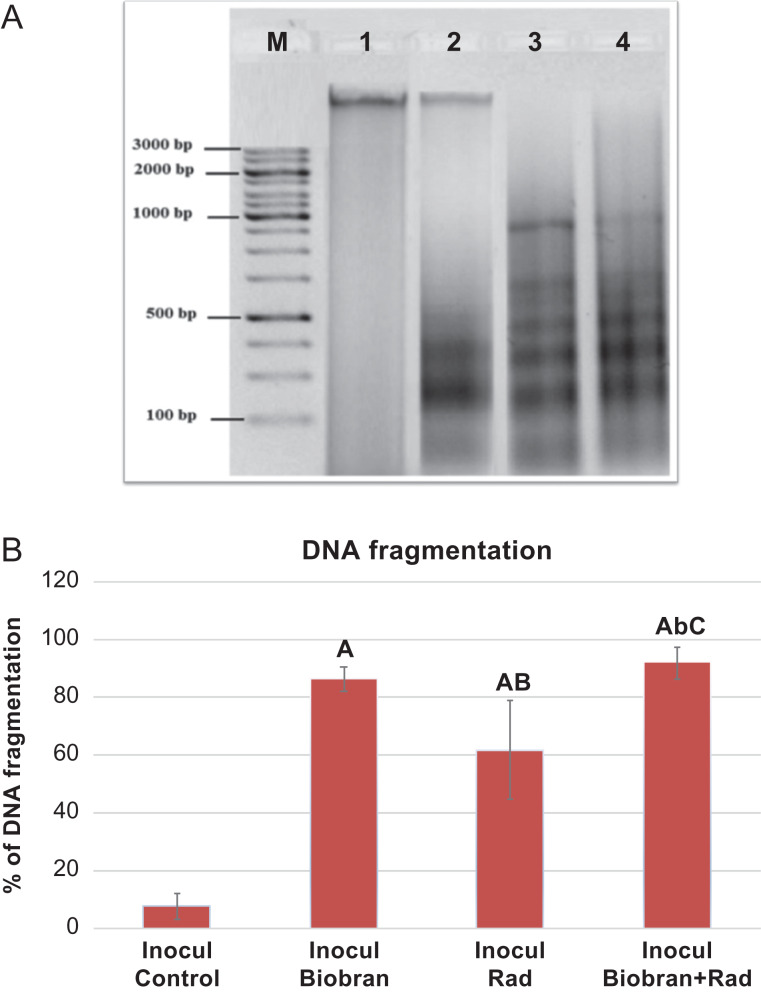
(A) Gel electrophoresis analysis of genomic DNA fragmentation in tumor tissues treated with Biobran and/or X-ray irradiation. The different lanes profiling the genomic DNA on agarose gel. Lane M: 100 bp molecular weight DNA ladder marker, lane 1: mice bearing tumor without treatment, lane 2: mice bearing tumor treated with X-ray irradiation, lane 3: mice bearing tumor treated with Biobran, lane 4: mice bearing tumor treated with Biobran and X-ray irradiation. (B) Percentage of DNA fragmentation obtained by gel electrophoresis in tumor tissue of tumor-bearing mice treated with Biobran and/or X-ray irradiation and analyzed by Image J software. Data are expressed as the mean ± SE of three different gel electrophoresis runs. ^A^Significantly different from Inocul group at *P* ≤ 0.01 level. ^b,B^Significantly different from Inocul Biobran group at *P* ≤ 0.05, *P* ≤ 0.01 level, respectively. ^C^Significantly different from Inocul Rad group at *P* ≤0.01 level.

As shown in Fig. 7A and B, the control untreated tumor cells produced 7.6 ± 4.5% of DNA fragmentation and showed no ladder formation. Biobran treatment produced 86.2 ± 4.3% of DNA fragmentation, and exposure to Rad resulted in 61.6 ± 17.1% of broken DNA strands, while treatment with Biobran + Rad showed the highest percentage of DNA laddering (91.7 ± 5.6%).

### Effect of Biobran and/or Rad on organ weight (liver weight and spleen weight)

On day 30, liver and spleen samples from each of the experimental groups were excised and weighed. Changes in organ weights are shown in Table 5. Treatment with Biobran alone of normal animals showed insignificant change in liver weight (LW) relative to normal control animals. As compared with the normal control group, an increase in LW under different treatments showed the following: Inocul Control mice 70.0%, Biobran 14.3%, Rad 52.9%, and Biobran + Rad minimized the increase in LW to 16.4%.

**Table 5.** Effect of Biobran and/or X-ray irradiation on liver weight and spleen weight; each value represents the mean ± SE of the corresponding number of animals/group

Parameter	Normal control	Biobran	Inocul			
			Control	Biobran	Rad	Biobran + Rad
**Liver weight**	**0.95 ± 0.07**	**1.03 ± 0.08**	**1.62 ± 0.07** *^,^**	**1.09 ± 0.06** ***	**1.46 ± 0.03** *^,^**^,‡^	**1.11 ± 0.09** ***^,¶^
**% Change from control group**	**-**	**+8.75**	**+69.96**	**+14.29**	**+52.9**	**+16.38**
**Spleen weight**	**0.19 ± 0.01**	**0.20 ± 0.01**	**0.32 ± 0.02** *^,^**	**0.21 ± 0.02** ***	**0.27 ± 0.02** *^,†^^,^***	**0.20 ± 0.02** ***^,¶^
**% Change from control group**	**-**	**+ 2. 0**	**+66.3**	**+7.5**	**+39.6**	**+3.7**
**Number of animals/group**	**8**	**8**	**10**	**11**	**6**	**10**

*Significantly different from control group at *P* < 0.01 level.

^†,^**Significantly different from Biobran group at *P* < 0.05, *P* < 0.01 levels.

***Significantly different from Inocul Control group at *P* < 0.01 level.

^‡^Significantly different from Inocul Biobran group at *P* < 0.05 level.

^¶^Significantly different from Inocul Rad group at *P* < 0.01 level.

Inocul Control mice showed a remarkable increase in spleen weight (SW) relative to the normal control group. Treatment with Biobran alone or Rad alone showed significant decrease in the SW relative to the Inocul Control group. In contrast, the combination of Biobran + Rad showed SW comparable with the normal control group. No significant changes in SW were observed in normal mice treated with Biobran alone as compared with the normal control mice (Table 5).

### Liver enzymes

Data in Table 6 represent the activity levels of liver function enzymes AST, ALT and GGT in serum of mice under different experimental conditions. Inocul Control mice showed marked increases in AST (165.7%), ALT (214.2%) and GGT (186%) levels as compared with the normal control group. Rad exposure recorded a significant elevation in liver enzymes AST, ALT and GGT levels to 189.8, 234.4 and 234.4%, respectively, as compared with the normal control group. Administration of Biobran improved the liver function by inducing remarkable reduction in the elevated AST, ALT and GGT levels to 86.8, 137.6 and 108.6%, respectively, as compared with the normal control group. Biobran + Rad significantly minimized the elevation of AST, ALT and GGT levels to 44.4, 97.2 and 71.8%, respectively, as compared with the normal control group.

**Table 6.** Effect of Biobran and/or X-ray irradiation on liver function tests; each value represents the mean ± SE of 5 samples/group.

Parameter	Normal Control	Inocul			
		Control	Biobran	Rad	Biobran + Rad
**AST(U/L)**	**59 ± 2.34**	**156.8 ± 5.68** *	**110.2 ± 4.78** *^,^**	**171 ± 6.4** *^,^***	**85.2 ± 2.85** *^,^**^,^***^,‡^
**% Change from normal control group**	**-**	**+165.7**	**+86.77**	**+189.8**	**+44.4**
**ALT(U/L)**	**36.2 ± 3.07**	**114 ± 5.75** *	**86 ± 2.56** *^,^**	**121 ± 5.93** *^,^***	**71.4 ± 2.80** *^,^**^,†,‡^
**% Change from normal control group**	**-**	**+214.2**	**+137.6**	**+234.4**	**+97.2**
**GGT(U/L)**	**26.08 ± 1.59**	**74.6 ± 4.73** *	**54.4 ± 3.62** *^,^**	**79.7 ± 4.97** *^,^***	**44.8 ± 3.00** *^,^**^,‡^
**% Change from normal control group**	**-**	**+186.0**	**+108.6**	**+205.6**	**+71.8**

*Significantly different from control group at *P* < 0.01 level, respectively.

**Significantly different from Inocul Control group at *P* < 0.01 level, respectively.

^†,^***Significantly different from Inocul Biobran group at *P* < 0.05, *P* < 0.01 levels^,^ respectively.

^‡^Significantly different from Inocul Rad group at *P* < 0.01 level^,^ respectively.

## DISCUSSION

Since radiation remains an important modality for cancer treatment, minimizing radiation therapy related toxicities has become a top priority. Enhancing the radioresponsiveness of tumors by using natural radiosensitizer agents has been shown to be a promising approach to improve the efficacy of radiation therapy, and this led us in the current study to investigate the potential beneficial effects of Biobran when used in concert with radiotherapy. Results of this study show that while treatment with either Biobran or Rad alone was effective in inhibiting tumor growth (volume and weight), combined treatment (Biobran + Rad) resulted in greater tumor regression. In our examination of the mechanism of Biobran’s action, the analysis of cell cycle progression showed that the combined treatment significantly increased the accumulation of hypodiploid cells in the sub-G1 phase. Sub-G1 cell accumulation signifies apoptosis, and for the combined treatment, the accumulation reached 123% relative to the control (*P* < 0.01), an increase that was higher than with either treatment alone. This increase did not result in a significant arrest of other cell cycle phases. Furthermore, the combined treatment increased the AI/PrI ratio 2.2-fold relative to control, while treatment with Rad alone only increased the ratio 1.5-fold.

DNA gel-electrophoresis showed that DNA damage of tumor tissues treated with Biobran + Rad showed the highest degree of DNA laddering. Annexin V/PI double staining also revealed that the combined treatment with Biobran + Rad exhibited greater induction of early apoptosis and a profound inhibition in the viable cell population, higher than with either treatment alone (data not shown). Several studies have shown that induction of apoptosis in cancer cells plays an important role in the efficacy of radiation therapy, and it has been considered the primary mode of radiation-induced regulated cell death. Radiation can directly affect the structure of the DNA double helix, which in turn activates DNA damage sensors to induce apoptosis and necrosis. The responses of tumor cells to heavy radiation-induced DNA damage are transmitted via DNA damage sensors and cell cycle regulators and can be categorized into three stages: DNA damage induction, DNA damage signal pathway activation, and DNA damage repair [27, 28].

The ability of Biobran to enhance the response of cancer cells to radiotherapy was also examined at the molecular level. RT-PCR and flow cytometry analysis revealed that treatment with Biobran + Rad significantly upregulated the expression of pro-apoptotic genes p53 and Bax and down-regulated the expression of the anti-apoptotic gene Bcl-2 to much greater extents than for treatments with either Biobran or Rad alone. Furthermore, the combined treatment maximized the percentage ratio of Bax to Bcl-2 protein expression in tumor cells. The overexpression of Bax, as well as of p53, is associated with the synergistic effect of Biobran on cancer cells. This corroborates previous reports of treating cancer cells *in vitro* and *in vivo* with gossypol or curcumin plus Rad, where the combination therapy showed the greatest increase in apoptotic cells [14, 29]. Other studies have also revealed a direct correlation between the degree of induced apoptosis and the cell response to irradiation [30]. Our results demonstrate that the extent of Rad’s apoptotic effect in cancer cells is significantly enhanced by adding Biobran treatment.

The Bcl-2 family proteins that consist of anti-apoptotic and pro-apoptotic members determine life-or-death of a cell [31]. Maximizing the percentage ratio of Bax to Bcl-2 protein expression in tumor cells from combined treatment (Biobran + Rad) resulted in activation of caspase-3 pathway. In the current study, the caspase-3 protein level was significantly upregulated post-treatment with Biobran + Rad to a higher level than with Rad treatment alone, indicating that Biobran-induced apoptosis occurs via a mitochondrial pathway. Biobran’s role in the activation of caspases-3, 8 and 9 and in the induction of apoptosis has previously been demonstrated [23]. Cleaved caspase-3 is considered to be a key executioner of apoptosis and essential for DNA fragmentation and chromatin condensation [32]. Therefore, Biobran’s potentiation of radiation therapy-induced apoptosis may be considered to be a result of Biobran’s enhancement of apoptotic regulators.

The protective role of Biobran against possible adverse effects induced by Rad and tumor burden was also studied here. The present results showed that treatment with Biobran, either alone or in combination with Rad, prevented BW loss and maintained liver and spleen weight close to normal values. These results are in agreement with earlier studies showing that Biobran provides protection against irradiation-induced BW loss [33] and acts as an adjuvant for chemotherapeutic drugs to maintain BW when combined with chemotherapy drugs such as paclitaxel, cisplatin or doxorubicin. [16, 34, 35]. The beneficial role of Biobran has been further confirmed in clinical trials of patients with hepatocellular carcinoma showing that Biobran treatment reduces cancer recurrence and prolongs life expectancy following chemotherapy [36]. The ability of Biobran to counteract the damaging effect of radiation is not fully understood, but could be attributed to the augmentory effects of Biobran on immune cells. Earlier studies have shown that Biobran is a potent biological response modifier known to activate dendritic cells [37, 38], enhance NK cell activity [19, 39], modulate cytokines and induce apoptosis in tumor tissue [15].

As a further radio-protective effect, our results also showed that treatment with Biobran, either alone or in combination with Rad, markedly decreased the elevated level of liver enzymes AST, ALT and GGT. This confirms Biobran’s protective role against radiation injuries to hepatocytes. Biobran has previously been shown to improve liver function as a result of its antioxidant activity, where it normalized lipid peroxidation levels, augmented glutathione contents and enhanced the activity of the antioxidant enzymes SOD, GPx, CAT and GST in the blood and liver of mice bearing tumors [40]. Whole body exposure to any form of radiation is known to alter the general physiology of an animal [41], and ionizing radiation inflicts its adverse effects through the generation of oxidative stress that unleashes large-scale destruction or damage of various biomolecules [42–44]. These free radicals react with body tissues and cause lipid peroxidation, DNA lesions and enzyme inactivation, all of which are mediators of radiation damage. Results of the current study correlate well with others who have reported that the sera of irradiated animals have elevated levels of AST, ALT and GGT [45, 46], an elevation which Biobran has here been shown to protect against.

In conclusion, this study demonstrates that MGN-3/Biobran, a natural and safe product extracted from rice bran, may serve as an agent for enhancing radiation-treatment efficacy. These effects may be achieved via the observed enhanced induction of tumor cell apoptosis, while also protecting body and organ weight and liver enzyme levels. These results suggest that Biobran may be used to potentiate the therapeutic effect of ionizing radiation in the treatment of solid tumors and to minimize its side effects on normal cells.
